# Retrospective OSL Dosimetry With Common Pharmaceuticals and Food Supplements

**DOI:** 10.3389/fpubh.2022.908016

**Published:** 2022-06-16

**Authors:** Daniela Ekendahl, Dan Reimitz

**Affiliations:** Department of Dosimetry, National Radiation Protection Institute, Prague, Czechia

**Keywords:** retrospective dosimetry, optically stimulated luminescence, radiation triage, pharmaceuticals, food supplements

## Abstract

Several common pharmaceuticals such as ibuprofen, paracetamol, aspirin, oral contraceptives, drugs for the prevention of motion sickness and food supplements such as table vitamins and minerals have been studied for the purposes of retrospective dosimetry using optically stimulated luminescence (OSL). The essence is that the tablets with these drug substances contain additive crystalline materials which, after irradiation and stimulation, may exhibit luminescence. For most of the pharmaceuticals and food supplements, a radiation-induced dose-dependent OSL signal was detected. Subsequently, basic dosimetric characteristics of the materials were studied, specifically sensitivity changes during repeated OSL readings, dose response, zero-dose, minimum detectable dose (MDD) and fading. The most radiation sensitive materials were food supplements with Mg providing zero-dose and MDD values at the level of several mGy. For Mg supplements, considerable sensitivity changes in OSL signal were observed. Despite this, they could be corrected using a Single-Aliquot Regenerative-dose (SAR) protocol. The OSL signals of the other materials were relatively weak but they were well reproducible and exhibited linear dose response. The MDD values were variable among the materials and ranged from 0.1 to several Gy. However, for some of the pharmaceuticals, a very high and variable zero-dose of more than 3 Gy was observed that would rule out the possibility of dose reconstruction for triage purposes. The OSL signal exhibited a significant fading rate for most of the materials. The measurements for dose reconstruction should be performed as soon as possible after irradiation, i.e. within a maximum of a few days.

## Introduction

The threat of serious radiation exposures to members of the public from radiological and nuclear incidents and accidents has led to intensive study of a number of emergency dosimetry methods for purposes of triage. The current methods of retrospective dosimetry include various biological, physical and computational techniques related to the radiation exposure of human body (1, 2). Among the physical techniques available, especially luminescence techniques using fortuitous materials and items of personal belongings are of interest. At least in Europe, the luminescence dosimetry instrumentation is relatively accessible. Research, mutual assistance, inter-laboratory comparisons, development and application of standardized methodologies are enhanced by the Running the European Network of Biological and retrospective Physical dosimetry (RENEB) association and European Radiation Dosimetry Group (EURADOS) activities (3, 4). It creates prerequisites for the possibility of rapid individualized dose estimations in cases of large-scale radiological events. Radiation sensitive components extracted from mobile phones and other small electronic devices are one of the most researched materials. Analytical protocols for dosimetry with alumina resistors and display glasses are available [e.g. (5–8)]. The luminescence techniques can also be applied for some biologically related materials such as dental ceramics and dental cement fillings (9, 10). Other potentially usable objects for luminescence techniques are banknotes, chip plastic cards, dust on coins, cigarette tobacco, fabrics and clothing, common household salt and salty products such as crackers and snacks, quartz extracted from bricks and other fired ceramic materials [e.g. (9, 11–17)].

Medications and food supplements can be included in the group of personal items. As regards their dosimetry potential, several studies were performed. Most of them were related to sterilization with gamma radiation and the physical methods used were electron paramagnetic resonance (EPR) or electron spin resonance (ESR) spectroscopy [e.g. (18–20)]. Only a few studies were conducted employing thermoluminescence (TL) or optically stimulated luminescence (OSL) (21, 22). The work of Sholom and McKeever (22) was related to emergency triage dosimetry and included both EPR and OSL measurements applied to a few food supplements such as multivitamins, minerals and amino-acids. Use of some common commercial drugs for post-sterilization and emergency dosimetry was studied in the work of Kazakis et al. (21), where TL and OSL signals were measured for doses from 20 to 1,400 Gy.

In this work, we investigated usability of pharmaceuticals and food supplements for retrospective dosimetry in the range of doses related to triage. The selection of the materials tested included common items such as ibuprofen, paracetamol, aspirin, oral contraceptives, drugs for the prevention of motion sickness, table vitamins and minerals, which people widely use in everyday life. The method of measurement was OSL. We focused on important characteristics of OSL signal of the samples such as sensitivity changes during repeated OSL readings, dose response, zero-dose, minimum detectable dose (MDD) and fading.

## Materials and Methods

### Preparation of Samples

The pharmaceuticals and food supplements used for this study were purchased at a pharmacy. They were tablets of different shapes and sizes. They were enclosed in common lightproof pharmaceutical packaging. Before the measurements, they were stored in the laboratory in the original packaging. The expiration date was not exceeded for any of the samples. In addition to the active pharmaceutical ingredients, the tablets contain excipients such as various antiadherents, binders, coatings, disintegrants, glidants and lubricants (23). They include crystalline materials such as talc, silica, titanium dioxide and magnesium oxide. The list of the pharmaceuticals used including information on their composition is given in Table 1. The food supplements with the details are listed in Table 2. All the data were taken from the patient information leaflets or packaging. The size and shape of most of the tablets in their intact form were not suitable for OSL measurement. It was necessary to adapt the material to the instrumentation and maximize the OSL yield. The tablets of all the materials were crushed using a mortar and a pestle. The powder was not sieved and thus contained grains of different size. Powder aliquots for measurements were created by covering cups used within the OSL instrumentation with a thin layer of the powder. The cups were discs with an inner diameter of 8 mm. The weight of the powder portions spread on the cups was 5 – 13 mg. The preparation was performed in darkroom conditions.

**Table 1:** List of the used pharmaceutical drugs and their composition. The data were taken from the patient information leaflets.

**Pharmaceutical**	**Brand name (manufacturer)**	**Active pharmaceutical ingredient**	**Excipients**
Aspirin	Acylpyrin (Herbacos Recordati s.r.o., Czechia)	Acetylsalicylic acid	Potato starch, talc
	Aspirin Protect 100 (Bayer AG, Germany)	Acetylsalicylic acid	Cellulose, corn starch, methacrylate copolymer, sodium laureth sulfate, Polysorbate 80, talc, triethyl citrate
Paracetamol	Panadol Extra (GlaxoSmithKline Dungarvan Ltd., Ireland)	Paracetamol, caffeine	Corn starch, pregelatinized starch, povidone, potassium sorbate, talc, stearic acid, sodium croscarmellose, hypromellose, triacetin
Ibuprofen	Ibuprofen (Medis International a.s., Czechia)	Isobutylphenylpropionic acid	Microcrystalline cellulose, colloidal anhydrous silica, hydroxypropyl cellulose, sodium laureth sulfate, sodium croscarmellose, talc, hypromellose, macrogol, titanium dioxide
	Nurofen (RB NL Brands B.V.,Netherlands)	Isobutylphenylpropionic acid	Sodium croscarmellose, sodium laureth sulfate, sodium citrate dehydrate, stearic acid, colloidal anhydrous silica, sodium croscarmellose, talc, gum arabic, sucrose, titanium dioxide, macrogol
Oral contraceptive	Birgi (Laboratorios León Farma, S.A., Spain)	Gestodene, ethinylestradiol	Lactose monohydrate, microcrystalline cellulose, magnesium stearate, polacrilin potassium, polyvinyl alcohol, titanium dioxide, soya lecithin, talc, iron oxide, xanthan gum
Drug for the prevention of motion sickness	Kinedryl (Noventis, s.r.o., Czechia)	Moxastine teoclate, anhydrous caffeine	Lactose monohydrate, corn starch, talc, calcium stearate

**Table 2:** List of the used food supplements and their composition. The data were taken from the patient information leaflets or packaging.

**Food supplement**	**Brand name (manufacturer)**	**Active pharmaceutical ingredient**	**Excipients**
Mixture of minerals and vitamins	Magne B6 Forte (Sanofi Winthrop Industrie, France)	Magnesium lactate dehydrate, pyridoxine hydrochloride	Sucrose, kaolinite, gum arabic, carbomer 934, talc, magnesium stearate, carnauba wax, titanium dioxide, iron oxide, propylene glycol
	Walmark Plus (Walmark, a.s., Czechia)	Calcium carbonate, magnesium oxide, zinc gluconate, magnesium gluconate, zinc citrate, calcium lactate gluconate, manganese gluconate, copper gluconate, cholecalciferol, pyridoxine hydroloride	Cellulose, silicon dioxide, talc, hypromellose, sodium carboxylmethylcellulose, titanium dioxide
Magnesium	Magnesium 1 (NatureVia/Swiss Natural, Canada)	Magnesium oxide	Microcrystalline cellulose, magnesium stearate, calcium phosphate, sodium carboxylmethylcellulose
Vitamin C	Celaskon (Zentiva, k.s., Slovakia)	Ascorbic acid	Corn starch, pregelatinized starch, edetate disodium dihydrate, talc, sodium stearyl fumarate, microcrystalline cellulose, sodium carboxylmethyl starch, beta-carotene powder

### OSL Instrumentation

OSL measurements were performed using an automated Risø TL/OSL reader model DA-20 equipped with an inbuilt ^90^Sr/^90^Y beta source of 1.48 GBq (2009-12-22). The optical stimulation was carried out with blue LEDs with a peak emission at 470 nm. The samples were stimulated and read using continuous-wave OSL (CW-OSL), in which the stimulation light intensity is kept constant and the OSL signal is monitored continuously throughout the stimulation period. The intensity of the light was 50 mW/cm^2^ at the sample position. The stimulation lasting 60 s was performed at room temperature. The luminescence light was detected using a system employing an EMI 9235QB photomultiplier tube and Hoya U340 glass filters. CW-OSL curves were recorded for all the aliquots measured. The OSL signal was quantified from the particular OSL curve so that the integral for the last 1.5 s was subtracted from the integral for the first 1.5 s. Dose rate in quartz provided by the inbuilt beta source was 0.081 Gy/s.

### Experiments

The experiments were focused on the main dosimetry characteristics as radiation sensitivity, zero-dose, minimum detectable dose (MDD), reproducibility, dose response and fading of the OSL signal. The purpose of the experiments was to find out the potential of the materials for dosimetry related to triage. For testing of reproducibility, dose response and fading, we repeatedly irradiated and read individual aliquots. The reason for this approach was that the amount of the radiation sensitive material can be limited in an emergency situation. In such cases, only a few aliquots would be available for dose reconstruction and thus a single-aliquot analytical protocol would be preferred. Alternative multi-aliquot protocols require enough material and time would not be feasible.

Our initial step was to investigate the radiation sensitivity of the pharmaceuticals and food supplements. Unirradiated aliquots of the materials were subjected to an initial OSL reading to find out whether they exhibit an initial luminescence signal. The purpose was to utilize the signal to determine zero-dose and MDD. Subsequently, the aliquots illuminated during the initial reading were irradiated to dose of 6.48 Gy to get an inkling of radiation sensitivity of the materials. The OSL signal obtained for each aliquot was normalized to its individual weight indicated by a high-precision scale (ED 224S-OCE, Sartorius AG, Germany) and to the dose applied. The measurement was performed with 12 aliquots of each material. In this way, average specific luminescence, c_specific_ (counts mg^−1^ Gy^−1^) was calculated for each material. The initial experiments included also measurements of irradiated aliquots that were not previously illuminated.

For the radiation sensitive materials exhibiting an obvious OSL signal, measurements of reproducibility, dose response and fading were performed using aliquots that were previously illuminated during the initial OSL reading. The individual aliquots of the materials were used repeatedly in several cycles included in the particular test. Each dosimetry characteristics was tested with at least 6 aliquots of the materials. All irradiations were made by means of the inbuilt beta source. The irradiated aliquots were kept in dark-room conditions inside of the reader between irradiation and reading.

To examine the reproducibility of the OSL signals was very important from the point of view of interpretation of the following dosimetry tests (dose response, fading) that were based on repeated use of individual aliquots. For the purpose of reproducibility measurement, aliquots of each material were irradiated and read in 10 identical cycles. The applied dose was 6.48 Gy. The OSL signal values in the cycles were normalized to the value obtained for the first cycle of measurement.

Dose response of the OSL signals of the materials was studied for doses of 0, 1, 2, 4, 6, 8, and 10 Gy. The dose responses obtained for the individual aliquots were corrected for their respective responses corresponding to dose D = 0 Gy that were measured by repeated readout following the initial readout. Data referred to D = 0 Gy thus differ from zero-dose data obtained by the initial readout. For materials with an unsatisfactory reproducibility (>10%), the dose response was measured using a Single-Aliquot Regenerative-dose (SAR) protocol that allows correcting the changes in sensitivity when the aliquot is used repeatedly (24). The SAR protocol included 7 cycles with the same regenerative doses as given above and a test dose of 0.8 Gy. For the aliquots used, dose response functions were derived. They provided the aliquot specific calibration to determine the zero-dose values based on the initial OSL signal measurement. The MDD was calculated taking three times the standard deviation of the zero-dose (16). It should be noticed that data

Fading of the OSL signal of the materials was studied for a period of 1 week. The aliquots were irradiated to dose of 6.48 Gy. The OSL readings were performed at different times post-irradiation, specifically 0.014, 0.1, 1, 5, 24, 72, and 168 h. Immediately after each OSL readout at time t_i_ providing response R(t_i_), an additional readout corresponding to the time point of 0.014 h for the same dose applied was performed providing response R(t_0_). Ratios of R(t_i_)/R(t_0_) were used for fading evaluation. The purpose was to reduce a possible influence of the reader sensitivity changes in time. In the case of materials with the unsatisfactory reproducibility, the evaluation was different. The responses R(t_i_) were determined using SAR protocol, that already provided an output unaffected by the reader sensitivity changes.

## Results and Discussion

### OSL Signal

**Figure 1:**
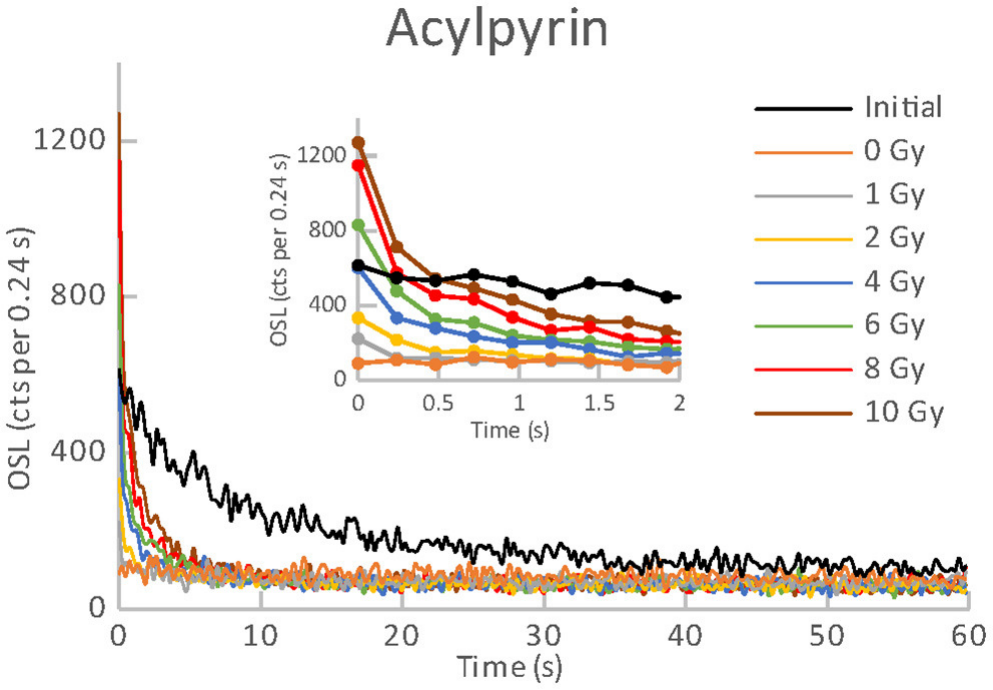
OSL curves for the initial readout and subsequent post-irradiations readouts of an aliquot of Acylpyrin.

**Figure 2:**
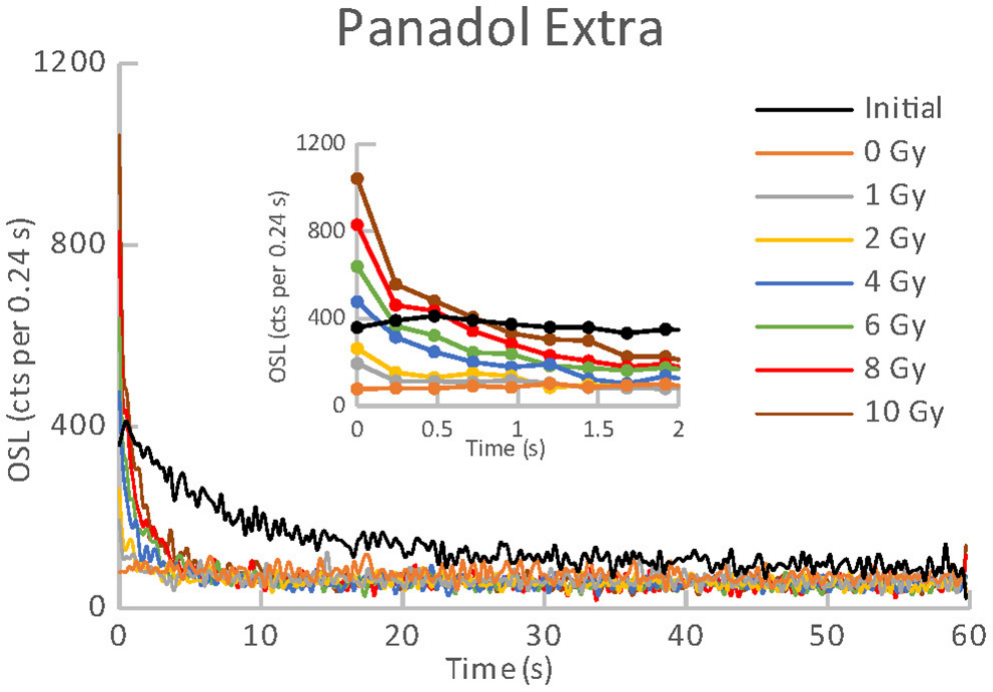
OSL curves for the initial readout and subsequent post-irradiations readouts of an aliquot of Panadol Extra.

**Figure 3:**
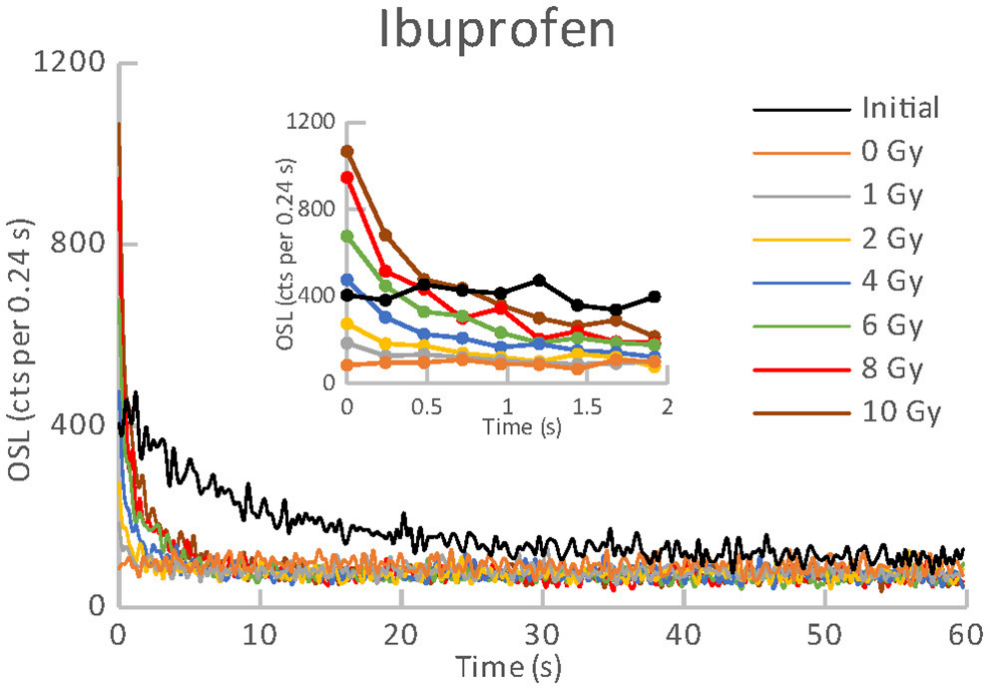
OSL curves for the initial readout and subsequent post-irradiations readouts of an aliquot of Ibuprofen.

**Figure 4:**
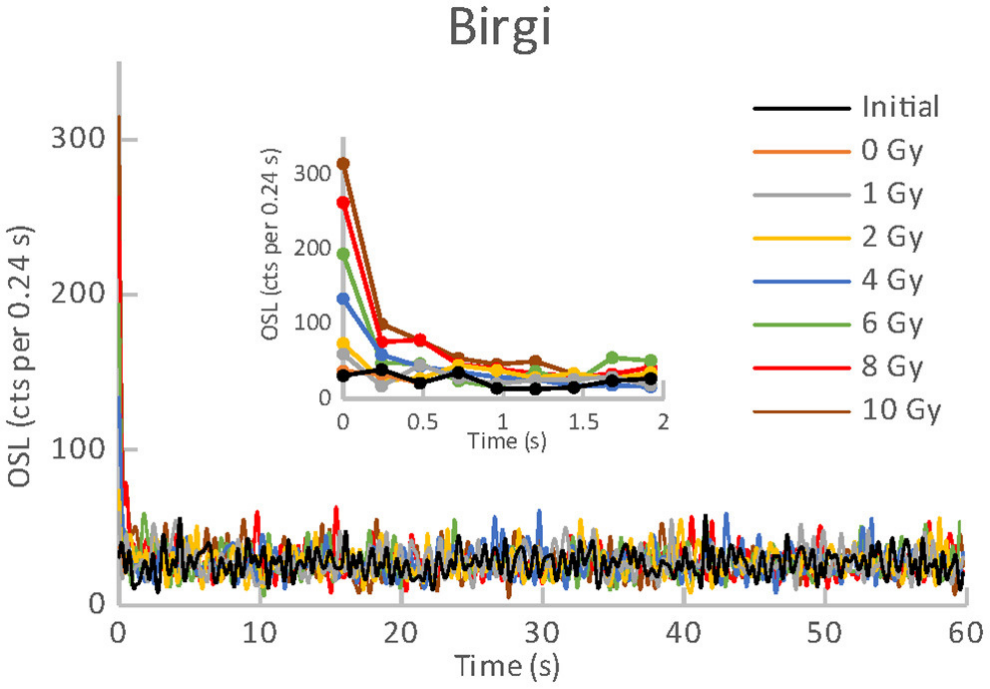
OSL curves for the initial readout and subsequent post-irradiations readouts of an aliquot of Birgi.

**Figure 5:**
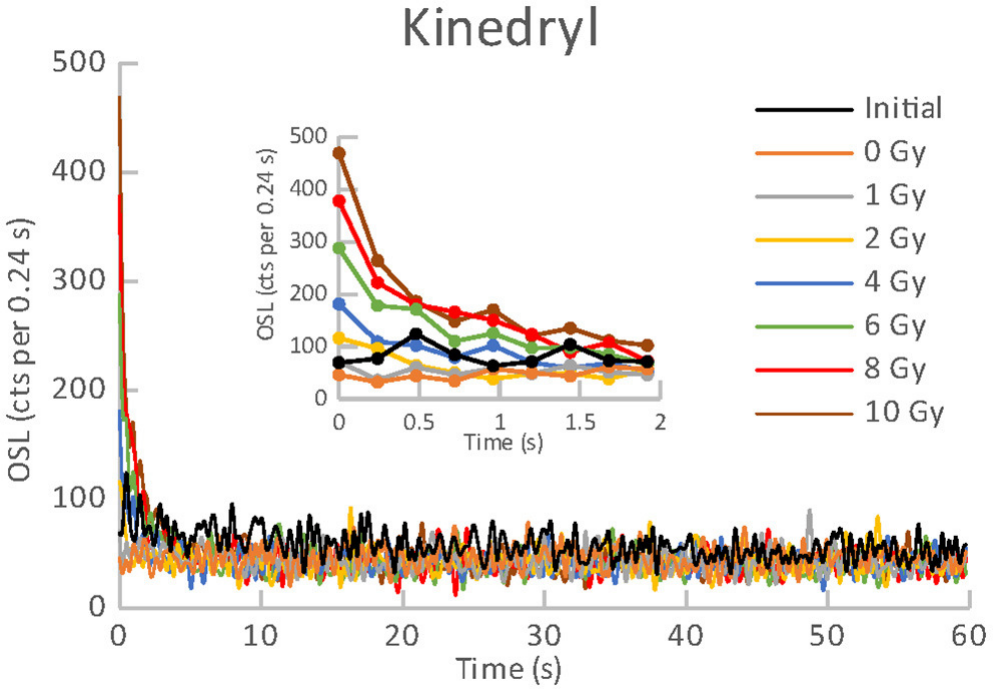
OSL curves for the initial readout and subsequent post-irradiations readouts of an aliquot of Kinedryl.

**Figure 6:**
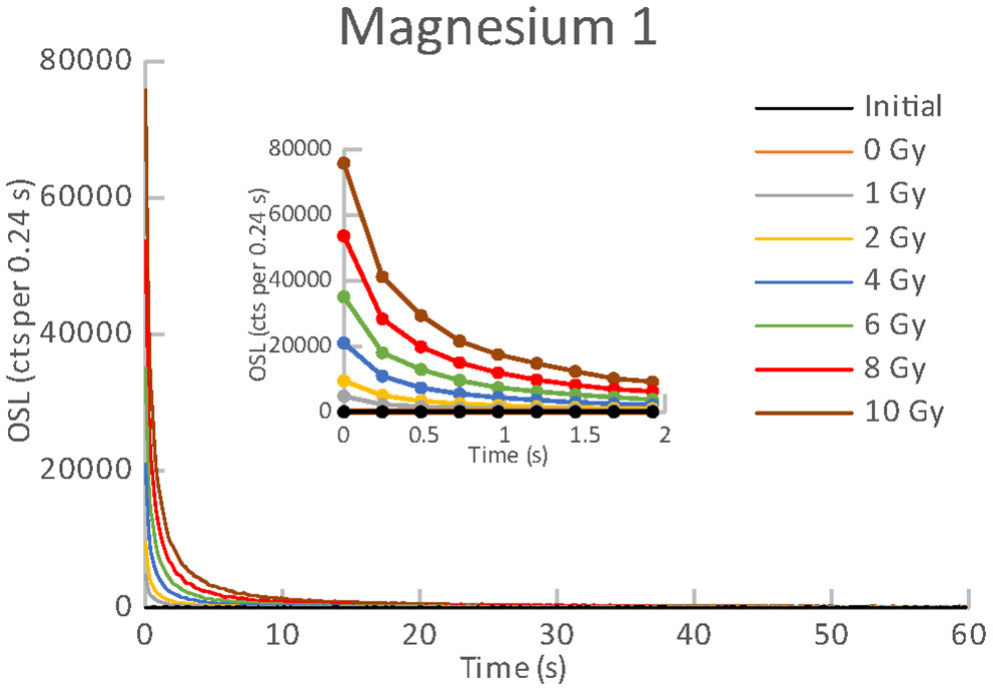
OSL curves for the initial readout and subsequent post-irradiations readouts of an aliquot of Magnesium 1.

**Figure 7:**
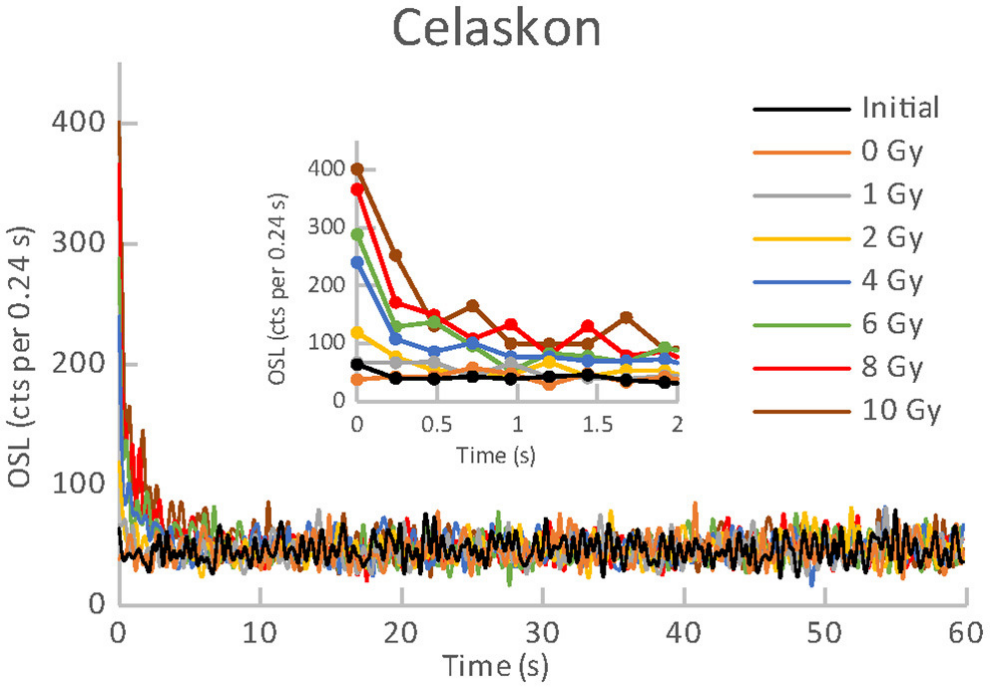
OSL curves for the initial readout and subsequent post-irradiations readouts of an aliquot of Celaskon.

All the materials listed in Tables 1, 2 exhibited an obvious OSL signal after illumination and test irradiation to dose of 6.48 Gy. The specific luminescence values c_specific_ (counts mg^−1^ Gy^−1^) are given in Table 3 for the particular materials. It is evident that the materials differ in radiation sensitivity. The most sensitive materials are the food supplements containing magnesium (Magnesium 1, Magne B6 Forte, Walmark Plus). It concerns especially Magnesium 1 that is a food supplement with predominating magnesium oxide. Magne B6 Forte containing magnesium lactate dehydrate instead of MgO is obviously less radiation sensitive. The OSL signal was relatively weak for all the pharmaceuticals (see Table 1), especially for Birgi and Kinedryl, and for vitamin C (Celaskon). OSL curves for one sample representing each pharmaceutical or food supplement are shown in Figures 1–7. Each of these figures includes OSL curves measured within the initial measurement and for doses up to 10 Gy subsequently applied. It should be noted that the “initial” measurement refers to OSL reading of an aliquot, which was not previously illuminated and irradiated, while the indication of “0 Gy” is related to OSL reading of the same already illuminated by the previous OSL reading and unirradiated aliquot. All the materials exhibit a dose dependent OSL signal with a rather fast decay. The insets in Figures 1–7 show the OSL signals during the first 2 s of the reading in detail. For some of the pharmaceuticals (Aspirin, Paracetamol, Ibuprofen) a distinct OSL signal was obtained within the initial measurement. This signal is characterized with a slower decay. Its source is unclear. It probably is of a non-ionizing radiation origin. At least in the Czech Republic, the common pharmaceuticals and food supplements are not sterilized with ionizing radiation. The occurrence of the initial OSL signal can possibly be ascribed to sunlight-UV irradiation or a specific illumination condition during the production. In the case of Aspirin, Paracetamol and Ibuprofen, the component of the slow decay was also marked for the aliquots that were irradiated, but not illuminated or read previously. The signal is depleted during the initial OSL reading and the following irradiations and readings provide OSL curves with a consistent form. The presence of the slow component of the OSL signal in the initial measurement can be used as a marker of a significant zero-dose of non-ionizing radiation origin. Such a strong initial OSL signal was not observed for samples of Aspirin, Ibuprofen and Paracetamol stimulated with blue light in the work of Kazakis et al. (21). For the other pharmaceuticals and food supplements, the initial signal is less distinct (Kinedryl, Celaskon) or hardly distinguishable from the subsequently measured signal corresponding to zero applied dose (Birgi, Magne B6 Forte, Walmark Plus, Magnesium 1).

**Table 3:** Specific luminescence, zero-dose and MDD values of all the materials.

**Sample**	**Specific lumimescence, c_**specific**_ (counts mg^**−1**^ Gy^**−1**^)**	**Zero-dose (Gy)**	**MDD (Gy)**
Acylpyrin	69 ± 13	8.35 ± 2.99	8.97
Aspirin Protect 100	35 ± 8	9.20 ± 2.51	7.54
Birgi	9 ± 2	0.35 ± 0.18	0.54
Celaskon	6 ± 1	0.15 ± 0.06	0.18
Ibuprofen	83 ± 15	10.12 ± 2.72	8.16
Kinedryl	7 ± 2	2.53 ± 1.13	3.39
Magne B6 Forte	156 ± 19	0.24 ± 0.06	0.18
Magnesium 1	2363 ± 213	0.05 ± 0.03	0.09
Nurofen	71 ± 13	10.73 ± 2.18	6.54
Panadol Extra	47 ± 9	6.23 ± 1.86	5.58
Walmark Plus	781 ± 126	0.06 ± 0.04	0.12

### Reproducibility

**Figure 8:**
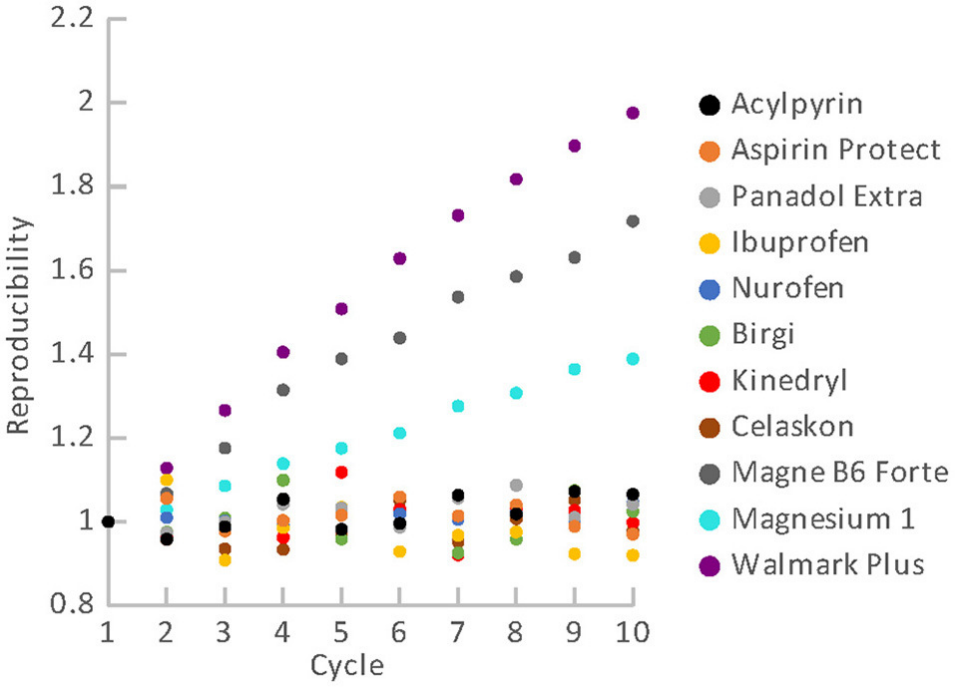
Reproducibility of OSL signal for the pharmaceuticals and food supplements.

Results of reproducibility measurements for all the used materials are shown in Figure 8. It is evident that the OSL signals of all the pharmaceuticals (see Table 1) and vitamin C (Celaskon) exhibit a satisfactory reproducibility (<10%). For these materials, the values of the normalized OSL signal oscillate around 1. It indicates that reproducibility should not significantly affect dose response of the OSL signal if the aliquot is used repeatedly. In contrast, the OSL signals of the food supplements containing magnesium (Magne B6 Forte, Walmark Plus, Magnesium 1) exhibit significant changes in sensitivity during the repeated cycles of irradiation and reading. There is an obvious gradual sensitization with the increasing number of cycles. It suggests that dose response of these materials would be supralinear, if any protocol correcting sensitivity changes was not applied.

In general, OSL sensitivity changes are usually caused by thermal and optical treatments of the material. The treatments can lead to thermo- and photo-transfers of charge carriers between traps (24, 25). In this case, the photo-transfers represent the more likely cause of the sensitization because all the OSL readings were performed at room temperature.

### Dose-Response, Zero-Dose and MDD

**Figure 9:**
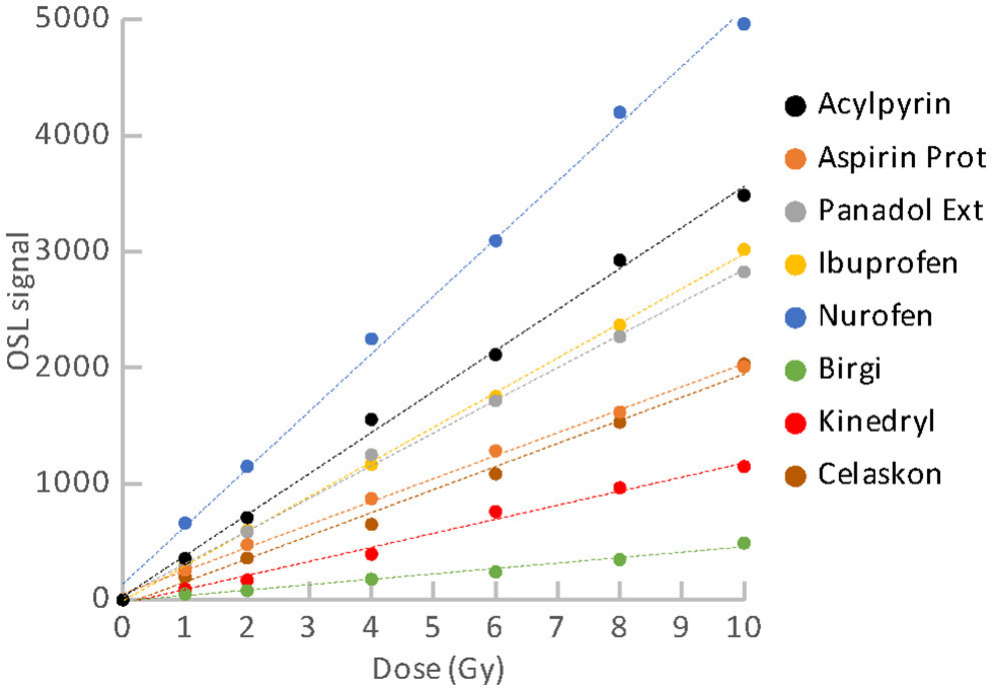
Dose response for the group of pharmaceuticals and vitamin C.

**Figure 10:**
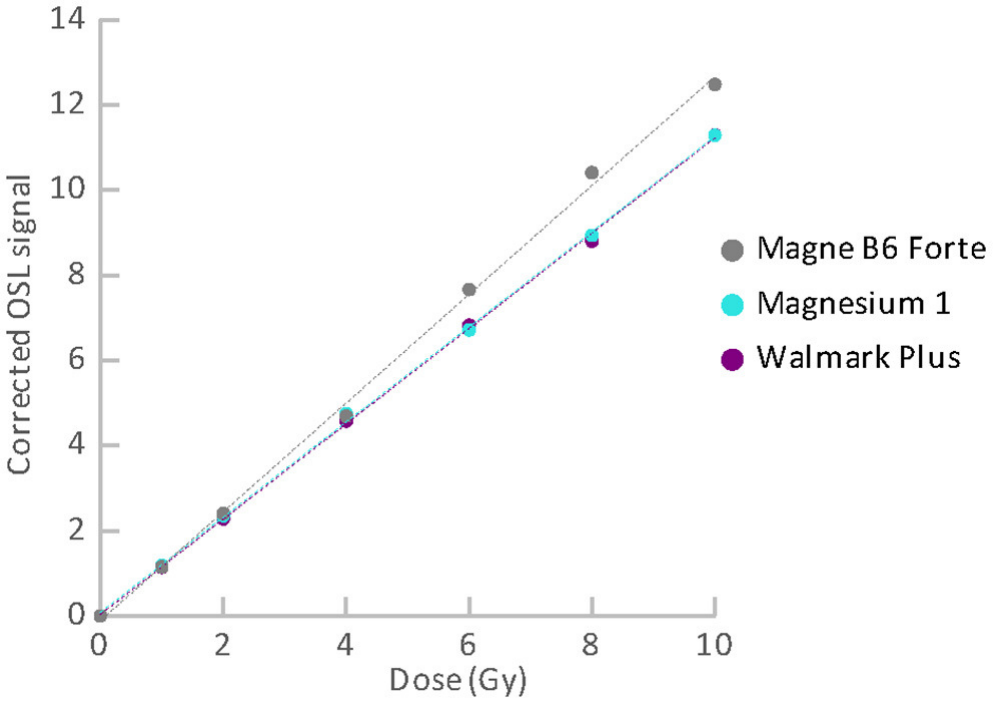
Dose response for the group of food supplements with Mg measured using SAR protocol.

Dose responses of the materials are illustrated in Figures 9, 10. Figure 9 refers to the materials with the satisfactory reproducibility, for which the dose response was measured in simple cycles of irradiation and reading. All these materials exhibit linear dose response. Results for the food supplements with magnesium that were obtained using the SAR protocol are shown in Figure 10. Their OSL signals corrected for sensitivity changes also exhibit linear dose dependence. The linear dose response for a few different mixtures of minerals (containing Mg) and vitamins was also observed in the previous study, in which the SAR protocol was applied (22). The authors mentioned that the SAR protocol worked well only for measurements without a temperature treatment.

Although we determined specific luminescence values for the materials (see Table 3) and all the aliquots provided OSL signals with linear dose dependence, we did not considered use of material specific universal calibration curves for dose reconstruction. The reason is that it cannot be assumed with certainty that different batches of the same material will behave in the same way. Besides, parameters of the linear function are not exactly the same for different aliquots of the same material. They may differ in dependence on amount of the powder, homogeneity of the powder and distribution of the powder on the cup. Consequently, dose reconstruction for purposes of triage should be based on construction of a dose response function for individual aliquots of the materials. The function enables to link the accident OSL signal with the corresponding accident dose. Because the OSL signals exhibit linear dose dependence in the range of doses related to triage, the analytical protocol can consist only of a few cycles of the measurement. In the case of the materials with good reproducibility, two simple cycles can be sufficient. The first readout will provide the “accidental” signal, while the second one will provide the calibration signal. For the materials with poor reproducibility (supplements with Mg), the SAR protocol must be applied. The protocol includes cycles of reading and irradiation for applied regenerative doses and a test dose. The measurement is more time demanding, but dose evaluation is very easy and quick, because the technique is implemented in the Analyst software, which is a part of the common Risø TL/OSL systems (26). In an emergency, a short SAR protocol consisting of two cycles can be used. However, if enough time is available, it is advisable to perform more cycles of measurement to determine the dose response curve better. In analyzing the dose response data, we calculated values of normalized dose response function as an indicator of supralinearity or sublinearity (27). The OSL signal of some of the aliquots exhibited a slight tendency to supralinear or sublinear dose response. However, the occurrence observed was rather random than specific for the particular materials.

Zero-dose and MDD represent very limiting and important factors for the feasibility of the dose reconstruction. Results of zero-dose and MDD based on the measurements of the initial OSL signal are given in Table 3. All the pharmaceuticals except for the oral contraceptive (Birgi) exhibit very high zero-dose and MDD values (>1 Gy), some of them reach or exceed doses for lethal effects of ionizing radiation that makes these materials practically inapplicable for the purpose of triage. In contrast, all the food supplements (Magne B6 Forte, Walmark Plus, Magnesium 1, Celaskon) exhibit zero-doses and MDDs with values less than 200 mGy, which is favorable from the point of view of emergency dosimetry. If a previously untested material was used in an emergency situation, the real values zero-dose and MDD would not be known. The identification that the initial OSL signal includes a significant contribution related to zero-dose can be done by comparing the OSL curves obtained during the initial readout and the following laboratory post-irradiation readouts. If the initial OSL curve is characterized by a markedly slower decay (as shown in Figures 1–3), the zero-dose will most likely be significant, and the determination of the ionizing radiation dose will not be possible.

### Fading

**Figure 11:**
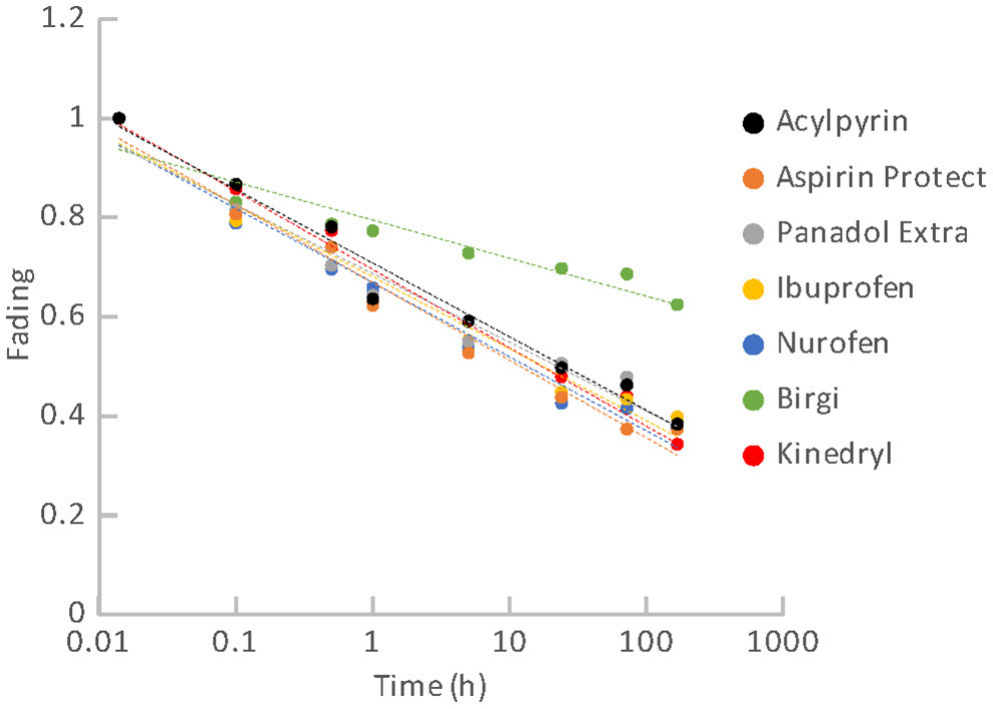
Fading for the group of pharmaceuticals.

**Figure 12:**
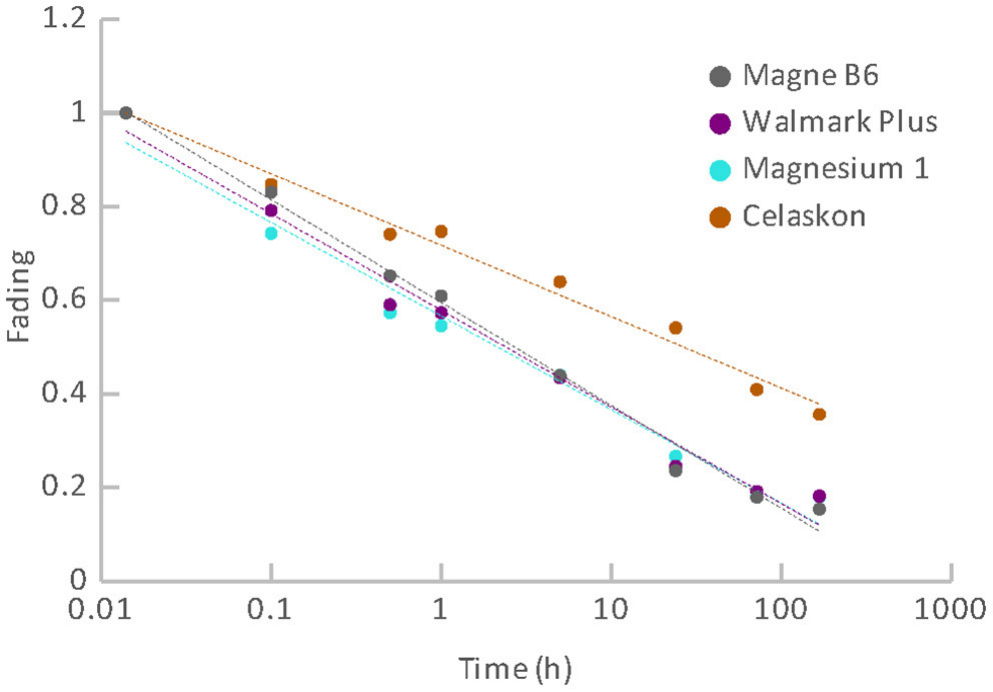
Fading for the group of food supplements.

Fading represents another important characteristic that can influence the possibility of dose reconstruction for triage purposes. Results of fading for groups of the materials are illustrated in Figures 11, 12. Figure 11 comprises results of fading of the samples of the pharmaceuticals group. Figure 12 includes results for the group of food supplements. It is evident that all the materials exhibit significant fading rates that can be described by a logarithmic function dependent on the time elapsed from the radiation exposure. Fading functions of all the pharmaceuticals except for the oral contraceptive (Birgi) exhibit a very similar trend. The greatest decrease of the OSL signals occurs during the first hours after irradiation. After 1 day the OSL signals fade to 43 – 50% of their original value. During 1 week, the signals decrease below 40% of their original value. The fading rate of Birgi is slower. The OSL signal drop reaches approximately 70% of its original value after 1 day and 62% after 1 week. As regards the food supplements, their OSL signals also exhibit a significant fading rate. It is considerable especially for the supplements with magnesium. The decrease of their OSL signals is very steep, after 1 day the signals fade to approximately 25% of the initial value. After 1 week, the signals drop below 20% of the original value. Fading for vitamin C (Celaskon) is rather similar to fading of the pharmaceuticals.

The fading observed can significantly impair the feasibility of dose reconstruction. It influences the realistic MDD value. The MDD values given in Table 3 are related to the time immediately after irradiation. For first dose estimate, the fading correction can be derived using the functions shown in Figures 11, 12. An aliquot specific fading correction can be derived based on repeated irradiations and measurements of the aliquot at times corresponding to a real time gap between the radiation exposure and the laboratory dose reconstruction, however, it would mean a delay. The dose reconstruction for triage purposes should be done as soon as possible after the radiation exposure.

A significant fading rate usually occurs for materials, for which the OSL signal is generated as a consequence of a charge carriers release from unstable shallow traps. To reduce the fading rate, pre-heating the aliquots before the OSL readout can remove the unstable components of OSL signal. For multivitamins and minerals (including Mg), the fading rate was significantly reduced by application of a pre-heat between 100 and 120°C (22). The residual OSL signal was still enough to reconstruct doses as low as a few hundreds of mGy, but it was also found that the SAR protocol stopped working due to temperature-related sensitivity change. Due to this fact, a multiple aliquot protocol was employed for the following measurements. Fading was monitored in the course of 7 days. After one week, the OSL signal decreased to approximately 70% of its initial value that was related to measurement immediately after irradiation. However, the thermal treatment is not always usable because different pharmaceuticals exhibit different heat resistance. Heating can cause their degradation and destruction (21). Consequently, the application of a thermal treatment is questionable.

### Further Discussion

The pharmaceuticals and food supplements are mixtures of various substances, and the mechanism of the OSL phenomenon is difficult to clarify. The crystalline substances occurring in these materials are the probable source of their OSL signal. One of them is talc, Mg_3_Si_4_O_10_(OH)_2_, which was mentioned as an excipient in all of the pharmaceuticals. Talc exhibits luminescence. TL of talc was measured in relation to the possible impact of talc on detection of irradiated foods (28). Other crystalline substances occuring in some of the materials are magnesium oxide and titanium dioxide. MgO and TiO_2_ doped with different elements were studied as luminescence detectors [e.g. (29–32)]. MgO probably plays very important role as a source of the OSL signal, because the OSL signal was the strongest for Magnesium 1 (see Table 2), in which MgO is the major component.

The general findings obtained in this study are restrained by the limited selection of the materials and should not be interpreted as meaning that all pharmaceuticals and food supplements in tablet form exhibit OSL after irradiation with doses <10 Gy. We initially tested samples of more brands of the common pharmaceuticals and food supplements, but some of them exhibited no OSL signal or a weak OSL signal that was distinguishable for doses higher than 10 Gy.

All the tablets used were white. It should be noticed that in the case of the oral contraceptive (Birgi), the blister pack contains 24 tablets with the active pharmaceutical ingredient, which are ochre, and 4 white inactive tablets. The tablets used for the OSL measurements were the inactive. We preferred white tablets because the presence of dyes could reduce the luminescence yield.

Compared to the other common materials used for retrospective dosimetry (e.g. alumina resistors, display glasses, chip modules, salt, quartz, dental ceramics), the pharmaceuticals exhibit a relatively low radiation sensitivity accompanied with very high zero-dose and MDD values (1). In contrast, the food supplements with Mg provide similar radiation sensitivity, zero-dose and MDD compared to the other retrospective dosimetry materials. A common problem is fading. Without a thermal treatment, similar high fading rates were also observed for other items usable in retrospective dosimetry, e.g. alumina resistors, chip modules, dental repair materials (8, 9). An advantage of drug tablets over the other retrospective dosimetry items is easy and fast preparation of aliquots for measurement.

In this study, we have not investigated a possible influence of optical bleaching. All the tablets used were removed from the original folding cartons or plastic non-transparent bottles in the darkroom conditions. Exposing the materials to daylight or laboratory light can lead to a partial loss of the OSL signal if the aliquots include the surface layer of the tablet. It can represent a practical problem because people usually handle the drugs and food supplements in the light. However, the surface layer can be removed by abrasion, at least in the case of bulkier tablets.

## Conclusion

Results of this study show that some of common pharmaceuticals provide measurable and satisfactorily reproducible OSL signal after irradiation. The signal is rather weak but increases linearly with dose in the range of doses related to triage. However, in the case of the drugs such as the tested brands of aspirin, paracetamol and ibuprofen, the OSL signal is not useful for dose reconstruction because of the high values of zero-dose and MDD. On the other hand, there exist common pharmaceuticals that have better potential for emergency dosimetry in the range of doses up to 10 Gy. One such example is the oral contraceptive tested with relatively low values of zero-dose, MDD and favorable fading rate.

As regards the food supplements, the results indicate that tablets containing magnesium oxide provide a relatively strong OSL signal after irradiation. Dose reconstruction can be performed using SAR protocol. For these supplements, zero-dose and MDD values are favorable from the point of view of emergency dosimetry. A problem is the high fading rate, but the dose reconstruction would still be feasible for doses at the level of a few Gy if performed no later than 24–72 h post-irradiation. Under such conditions, the supplements with MgO offer similar advantages as other personal items suggested for luminescence emergency dosimetry.

## Data Availability Statement

The raw data supporting the conclusions of this article will be made available by the authors, without undue reservation.

## Author Contributions

DE: study concepts and design, measurement, data analysis and interpretation, and manuscript preparation. DR: preparation of samples, measurement, data processing, and preparation of tables and graphs. Both authors contributed to the paper and approved the submitted version.

## Funding

This work was supported by the Ministry of the Interior of the Czech Republic, identification code MV-17196-1/OBVV/2022.

## Conflict of Interest

The authors declare that the research was conducted in the absence of any commercial or financial relationships that could be construed as a potential conflict of interest.

## Publisher's Note

All claims expressed in this article are solely those of the authors and do not necessarily represent those of their affiliated organizations, or those of the publisher, the editors and the reviewers. Any product that may be evaluated in this article, or claim that may be made by its manufacturer, is not guaranteed or endorsed by the publisher.
